# Editorial: Current advances in genetic dementia and aging

**DOI:** 10.3389/fnagi.2022.1020547

**Published:** 2022-09-14

**Authors:** Yuzhen Xu, Daojun Hong, Ulises Gomez-Pinedo, Jun Liu, Jun Xu

**Affiliations:** ^1^School of Medicine, Tongji University, Shanghai, China; ^2^Department of Neurology, The First Affiliated Hospital of Nanchang University, Nanchang, China; ^3^Laboratory of Neurobiology, Department of Neurology, Institute of Neurosciences, IdISSC, Hospital Clínico San Carlos, Universidad Complutense de Madrid, Madrid, Spain; ^4^Department of Neurology, The Second Affiliated Hospital, Guangzhou Medical University, Guangzhou, China; ^5^Department of Neurology, Beijing Tiantan Hospital, Capital Medical University, Beijing, China; ^6^China National Clinical Research Center for Neurological Diseases, Beijing Tiantan Hospital, Capital Medical University, Beijing, China

**Keywords:** dementia, Alzheimer's disease (AD), polygenic, neuroimaging, Aβ/tau

Cognitive impairment and dementia have become a serious global public-health issue associated with severe disability. Alzheimer's disease (AD) is a multifarious polygenic disease that is the most common cause of dementia in the elderly. Early-onset AD (EOAD) accounts for approximately 5–10% of all cases of AD and can be caused by mutations in amyloid precursor protein (APP), presenilin 1 (PSEN1), and presenilin 2 (PSEN2). According to Orozco-Barajas et al. PSEN1 A431E is the most reported variants related to EOAD. Besides, more than 40 other genetic variants combine to cause late-onset AD (LOAD) with a lifetime prevalence of 22–95%; when demographic factors are included in the risk score along with APOE ε4 and other risk genes, AD prediction accuracy will exceed 66% (Koriath et al., 2021). And other APOE ε4-independent LOAD, with the lowest risk of development, is significantly more likely to be caused by random conditions than by genetic factors. Previous studies have shown that patients with cerebrovascular illness are more likely to develop dementia. The role of modifiable vascular risk factors can play a role, such as hypertension, diabetes, hyperlipidemia and so on (Appleton et al., 2017; Yen et al., 2022). Strategies to prevent cerebrovascular illness hold great potential to delay the incidence of cognitive impairment or dementia.

Neuroimaging advancements could help in better understanding of AD neuropathologic changes *in vivo*. Zeng et al. have found that vascular hyperintensity on FLAIR images has important implications for cerebrovascular disease, for instance, large-vessel stenosis/occlusion, Moyamoya disease and transient ischemic attack, but it cannot be used as a neuroimaging marker of impaired cerebral hemodynamics or good collateral circulation. Resting-state functional magnetic resonance imaging (rs-fMRI) researches have found that individuals with age-related macular degeneration exhibit abnormal functional connection and regional homogeneity in Liu et al. and Xiao et al. In addition, patients with mild cognitive impairment have regional homogeneity dysfunction, but their signal intensity in several brain regions is higher than in normal controls. These results may be related to the neural network excitation and inhibition of different regions (Wu et al.). Mai et al. have found that an AD-resemblance atrophy index, based on structural magnetic resonance imaging, can be used to predict the risk of progression from MCI to AD dementia. It is also beneficial to construct a more accurate risk prediction model for AD.

Polygenic risk score combined small effect SNPs is a useful approach to identify individuals at higher risk of LOAD. Mendelian randomization studies have found that AD genetic susceptibility is negatively associated with gout, but not be causally, and genetic associations between LOAD and other cardiometabolic risk factors have been reported (Fu et al.; Li et al.). Increasing attention is now being directed to intestinal flora, Zhang et al. have found that significantly greater numbers of genus Helicobacter is observed in diabetic mice than in wild mice and diabetes may be intricately linked to increased risk for developing neuro-inflammation, which potentially induce age-related cognitive impairment.

The amyloid hypothesis is widely accepted as the core pathology of AD, and amyloid-beta deposition is posited to be the initiating factor in AD. Amyloid-beta may suppress the expression of SIRT1, an essential aging regulator, and promote aging-associated DNA damage. Interestingly, Aspirin could upregulate SIRT1 expression and rescue cells from senescence (Li et al.). Moreover, the accumulation of tau neurofibrillary tangles is a major pathological hallmark of AD. Qian et al. have proved that KRAS and PIK3R1 gene, as core genes associated with abnormal infiltration of peripheral immune cells, are strongly associated with the severity of tau pathology.

Many attempts have been made to prevent or postpone dementia progression, such as exercise, nutritional supplementation and medications. Chen et al. indicate that exercise may be significant to prevent AD through upregulating the expression profile of miR-215-5p to prevent neuronal cell necrosis. Based on molecular dynamical simulations and kinetic studies, Kang et al. confirm that Baicalein can contribute to protect patients with atrophic rhinitis against the development and progression of MCI by targeting the cytochrome C protein.

## Author contributions

JX composed the main outline of the editorial. YX drafted the manuscript. All authors contributed to the article and approved the submitted version.

## Funding

This study was supported by the National Key Research and Development Program of China (2021YFC2500103), the National Natural Science Foundation (Grant Nos. 82071187, 81870821, and 81471215), and Beijing Youth Talent Team Support Program (2018000021223TD08).

## Conflict of interest

The authors declare that the research was conducted in the absence of any commercial or financial relationships that could be construed as a potential conflict of interest.

## Publisher's note

All claims expressed in this article are solely those of the authors and do not necessarily represent those of their affiliated organizations, or those of the publisher, the editors and the reviewers. Any product that may be evaluated in this article, or claim that may be made by its manufacturer, is not guaranteed or endorsed by the publisher.
